# miRNA-221-3p derived from M2-polarized tumor-associated macrophage exosomes aggravates the growth and metastasis of osteosarcoma through SOCS3/JAK2/STAT3 axis

**DOI:** 10.18632/aging.203388

**Published:** 2021-08-13

**Authors:** Wei Liu, Qiuping Long, Wei Zhang, Dehui Zeng, Bingbing Hu, Shengyao Liu, Li Chen

**Affiliations:** 1 Department of Orthopedics Trauma, The Affiliated Nanhua Hospital, Hengyang Medical School, University of South China, Hengyang 421002, Hunan, China; 2 Department of Orthopedics, The Second Affiliated Hospital of Guangzhou Medical University, Guangzhou 511436, Guangdong, China

**Keywords:** miR-221-3p, exosome, tumor-associated macrophages, SOCS3, JAK2, STAT3, osteosarcoma

## Abstract

Background: Enhanced infiltration of M2-polarized tumor-associated macrophages (TAMs) is linked to osteosarcoma (OS) metastasis and growth. Here, we aim to explore a novel miR-221-3p shuttled by M2-TAM exosomes in the growth and metastasis of OS cells.

Methods: THP-1 monocytes-derived M2-TAMs were induced by PMA/interleukin (IL)-4/IL-13 and then co-cultured with OS 143B and Saos2 cells. Overexpression or downregulation models of miR-221-3p were conducted to probe the impacts of exosome-derived M2-TAMs in OS cells. OS cell proliferative ability, colony formation, invasion, migration and apoptotic level were measured by the cell counting kit-8 (CCK-8) assay, colony formation, Transwell assay, and flow cytometry. Moreover, the SOCS3/JAK2/STAT3 axis in OS cells was testified by western blot, and a dual-luciferase reporter assay was conducted to confirm the link between miR-221-3p and SOCS3.

Results: OS cells enhanced M2 polarization of TAMs, which significantly promoted OS cells’ viability, colony formation, migration, invasion, and reduced apoptosis. Moreover, the exosomes enriched by miR-221-3p from M2-polarized TAMs (M2-TAMs) also aggravated the malignant behaviors of OS cells. However, down-regulation of miR-221-3p brought about contrary results. Further, *in-vivo* tests uncovered that overexpressing miR-221-3p enhanced OS cells’ growth. Mechanistically, SOCS3 was a downstream target of miR-221-3p, and up-regulation of miR-221-3p choked SOCS3 and activated JAK2/STAT3. However, the pharmacological intervention of the JAK2/STAT3 pathway obviously inhibited the malignant behaviors of OS cells, which were significantly reversed by miR-221-3p up-regulation.

Conclusion: The exosomal miR-221-3p derived from M2-TAMs aggravates OS progression via modulating the SOCS3/JAK2/STAT3 axis.

## INTRODUCTION

Osteosarcoma (OS) is a kind of primary bone malignancy with immature bone tissue and osteoid produced by mesenchymal cells, which is most common in teenagers aged 15 years to 19 years old [1]. It is highly metastatic, with a high disability and mortality rate, which is prone to relapse and causes a dreadful prognosis [2]. OS lesions are mostly located in the long tubular bones, like the terminal femur and the upper tibia, as well as the fibula, spine, humerus, ilium, etc. [3]. Currently, it is generally believed that OS is a malignant tumor formed by the occurrence of gene mutations during the growth and differentiation of mesenchymal cells, which destroys normal bone development and leads to malignant changes in bone cells [4]. Therefore, it is crucial to identify new biomarkers for OS.

Presently, increasing studies have revealed that the tumor microenvironment (TME) affects development and progression. Tumor-associated macrophages (TAMs) are an important part of the TME in OS, accounting for over 50% of the immune cells. They have a wide range of biological functions, including inducing tumorigenesis, angiogenesis, immunosuppression, drug resistance and metastasis [5]. TAMs may contribute to the malignant progression of OS via self-polarization, the facilitation of vascular and lymphatic vessel formation, immunosuppression, and drug resistance [6]. Since the immune microenvironment of OS and Ewing's sarcoma signally affect patients’ prognosis, evaluation of immune infiltration is an effective method for diagnosing bone-associated malignancies [7]. Zheng et al. [8] found that the level of hub gene CD163 (the “M2” macrophages marker) was negatively associated with OS. In a review paper, Cersosimo et al. concluded that enhanced infiltration of M2-polarized tumor-associated macrophages (M2-TAMs) is related to OS metastasis and unsatisfactory prognosis regardless of currently adopted aggressive therapies regimens [9]. Therefore, M2-TAMs aggravate the malignant behaviors of OS cells. Besides, M2-TAM exosomes conduct intercellular communication in the tumor microenvironment, induce tube formation of vascular endothelial cells and aggravate OS progression via the genetic information carried by exosomes [10–11]. However, the association between M2-TAM exosomes and OS cell growth and development needs further exploration.

Previous reports have confirmed that tumor-derived exosomes carry microRNAs (miRNAs), proteins, lipid molecules, etc., which modulate OS cell growth and development [12]. miRNAs are approximately 22 nucleotides long, which modulate gene expression after transcription [13]. Multiple miRNAs are implicated in OS. For instance, miR-1225-5p, miR-139-5p, and miR-486 dampen the growth, invasion, and epithelial-mesenchymal transition (EMT) of OS cells and play a tumor-suppressive role [14–16]. On the contrary, Wang JW et al. claimed that tumor-associated fibroblasts (CAFs) transfers miR-1228 from exosomes to OS cells, which up-regulates miR-1228 in OS cells, down-regulates endogenous Suppressor of cancer cell invasion (SCAI), and strengthens OS cell migration and invasion [17]. Interestingly, previous research has claimed that hsa-miR-221-3p expression is heightened in small cell osteosarcoma (SCO), an infrequent subtype of OS, with a highly aggressive progression and unsatisfactory prognosis [18]. However, the effect of miR-221-3p derived from TAMs exosomes on OS cell growth and metastasis and its mechanism is still unclear.

In this experiment, we probed the effect of miR-221-3p in M2-TAM exosomes on OS cells and found that M2-TAMs facilitate OS cell growth, metastasis and invasion. And miR-221-3p shuttled by M2-TAM exosomes also aggravates OS. Meanwhile, miR-221-3p targets SOCS3 and up-regulates JAK2/STAT3. Therefore, we posit that miR-221-3p aggravates OS by regulating the SOCS3/JAK2/STAT3 axis. In conclusion, our experiment testifies that miR-221-3p is expected to be a novel target for treating OS.

## METHODS

### Cell culture

The Type Culture Collection of the Chinese Academy of Sciences (Shanghai, China) provided human OS cell lines (143B and Saos2) and mononuclear macrophages (THP-1). Cells were grown at 37°C with 5% CO_2_ in the RPMI 1640 medium consisting of 10% fetal bovine serum (FBS) and 1% penicillin/streptomycin (Invitrogen, CA, USA). The FBS and RPMI 1640 were commercially provided by Thermo Fisher Scientific (MA, USA). We changed the medium every fourth day. 0.25% trypsin (Thermo Fisher HyClone, Utah, USA) was used for the digestion of cells in the logarithmic growth stage for the following test.

### Cell transfection

Cells at the logarithmic growth stage were dispersed in 6-well plates (5 × 10^6^ cell/well) and transfected after they grew stable. The FuGENE^®^HD Transfection Reagent (Roche, Shanghai, China) was utilized to transfect miR-221-3p mimics, miRNA control fragments (miR-NC), miR-221-3p inhibitors (miR-221-3p-in), and negative controls (NC-in) into THP1 cells, respectively. Cells were incubated at 37°C with 5% CO_2_, and the stably transfected cells were taken after transfection for one day.

### M2 polarization of TAMs

1 × 10^7^ THP-1 cells were maintained in a 10 cm culture dish comprising 200 ng/mL PMA, and the medium was altered after one-day incubation. Then, THP-1 macrophages (M0) were collected. Afterward, M0 was incubated in 10 mL fresh complete medium. Meanwhile, 20 ng/mL of IL-4 and IL-13 (PeproTech) were added to the culture medium containing 143B and Saos2 cells for 72 hours. 10^6^ M2-TAMs were harvested. Subsequently, M2-TAMs were further grown in 10 mL serum-free RPP medium. The conditioned medium (CM) was centrifuged (1300 rpm, 5 min), and the supernatant was preserved at −80°C in equal portions.

### Isolation of TAM exosomes

THP1 cells were treated with IL4/IL-14 and then transfected with miR-221-3p mimics or miR-221-3p-in. Then the cells were seeded in 10-cm culture dishes. After a 48-hour culture with a fresh basic medium containing exosome-free serum, the culture medium of THP1 cells was harvested and ultra-centrifuged at 200 rpm for removing cell debris. Next, sequential differential centrifugation was conducted for the isolation of exosomes: 1000 rpm for 5 min, 2000 rpm for 20 min, 5000 rpm for 30 min, 10000 rpm for 30 min and 100000 rpm for 70 min. Finally, the exosomes were resuspended in PBS and centrifuged at 100000 rpm for 70 min. The isolated exosomes were identified by a field-emission scanning electron microscope (JEOL JSM-7100F/LV). The exosome markers, including CD63, CD9 and Tsg101 was determined by western blot (WB). The BSA method was employed for testing the concentration of exosomes.

### Quantitative reverse transcription-polymerase chain reaction (qRT-PCR)

The miR-221-3p expression in the co-culture model was measured with the TRIzol reagent (Invitrogen, Carlsbad, CA, USA). The PrimeScript™ RT Reagent kit (Invitrogen, Shanghai, China) was adopted for cDNA synthesis. qRT-PCR was conducted with the SYBR Green PCR Reagent and ABI7500FAST Real-Time PCR instrument. The 2^−ΔΔCt^ method was applied to assess the relative expression of miR-221-3p (U6 and GAPDH were endogenous control). The primer sequence is as follows:

**Table:** 

**The target**	**Forward (5′–3′)**	**Reverse (5′–3′)**
miR-221-3p	AACCGGAGCTACATTGTCTGCT	CAGTGCAGGGTCCGAGGT
IL-1β	TGACTGACTCTCCT	ACTGGGCACTCAATTCCA
IL-10	GCTCCCTGGTTTCTCTTCCT	GTTCTTTGGGGAGCCAACAG
Arg1	GCTTTTCCCACAGACCTTGG	GTGGAAGAAGGCCCTACAGT
YM1	CAAGTTGAAGGCTCAGTGGCTC	CAAATCATTGTGTAAAGCTCCTCTC
U6	CTCGCTTCGGCAGCACA	ACGCTTCACGAATTTGCGT
GAPDH	TGGTTGAGCACAGGGTACTT	CCAAGGAGTAAGACCCCTGG

### Cell counting kit-8 (CCK-8) assay

Cells at the logarithmic growth stage were trypsinized. They were then inoculated in 96-well plates (2 × 103/well), and each well was filled with 200 μL DMEM medium and cultured at 37°C with 5% CO_2_. When the cell abundance reached 50%, a fresh serum-free medium was employed to replace the primary one, and the serum was added after 24 hours. After 24 hours of culture, the CCK-8 solution (Beyotime Biotechnology, Shanghai, China) was supplemented (10 μ/well), and the absorbance value was observed at 450 nm.

### Colony formation assay

Cell colony formation was monitored by the cell colony formation assay. Cells in the logarithmic growth stage were dispersed in 6-well plates (1000 cells/well) and cultured at 37°C with 5% CO_2_. Fourteen days later, the culture medium was removed, and the cells were rinsed with PBS three times and fastened with 4% paraformaldehyde for 20 min. Then the cells were dyed with 1% methylene blue for 40 min, washed twice with deionized water, and dried. The stained colonies were imaged and counted using an optical microscope.

### Flow cytometry (FCM)

Cells treated by the above factors were harvested and rinsed with PBS twice. The cells were resuspended in a 150 μL binding buffer. The buffer was then filled with 10 μL Annexin V-FITC and 5 μL propidium iodide (PI). The cells were mixed with a centrifuge tube and incubated for 15 min at 4°C away from light. The apoptosis rate was verified by FCM according to the Annexin V-FITC/PI Apoptosis Detection Kit (Yeasen Biotech Co., Ltd.).

### Western blot

2 × 10^6^ cells of each group were cleaned three times with prechilled PBS and cleaved with the RIPA lysate, and the supernatant was collected. The protein content was calculated by the BCA method. After being subjected to polyacrylamide gel electrophoresis, 50 μg total protein was transferred to polyvinylidene fluoride (PVDF) membranes. Then, 5% skimmed milk was employed to seal the membranes (at room temperature, RT, 2 hours), which were then washed with 0.05% TBST three times and maintained with primary antibodies (concentration: 1:1000; Abcam, MA, USA) of Bax (ab32503), Bcl2 (ab218123), Cleaved Caspase-3 (ab32042), iNOS (ab178945), STAT6 (ab32520), SOCS3 (ab16030), JAK2 (ab108596), p-JAK2 (ab32101), STAT3 (ab68153), p-STAT3 (ab76315), and β-actin (ab115777) at 4°C overnight. After the membranes were washed with TBST, the HRP-labeled rabbit secondary antibody (1:300) was added and incubated for an hour. Next, the membranes were rinsed three times with TBST. At last, the WB special reagent (Invitrogen) was adopted for color development, and each protein’s gray intensity was evaluated by Image J.

### Transwell assay

Cell migration experiment: The above groups of cells at the logarithmic growth stage were put in the upper chamber of the Transwell (2 × 10^4^ cells/well), and a 600 μL culture medium comprising 20% FBS was added and cultured at 37°C in the lower chamber. After 12 hours, cells that failed to migrate were wiped off. The lower chamber cells were immobilized with 4% paraformaldehyde and dyed with 0.1% crystal violet. After drying, the cells were imaged and calculated. The cell invasion experiment was implemented as the migration assay except that the Transwell’s upper chamber was pre-coated with Matrigel. Cell migration and invasion were observed under an inverted microscope.

### Dual-luciferase reporter assay

TargetScan indicated that SOCS3 was an underlying target of miR-221-3p. The reporter plasmids of wild-type and mutant SOCS3 were designed. miR-221-3p mimics and their controls (miR-NC) were transfected into THP-1 cells. Two days later, the luciferase activity was evaluated as per the dual-luciferase reporter assay guidelines (Promega, Madison, WI, USA). All tests were made three times.

### Xenograft experiment

Twenty BALB/c mice (male, 4–8 weeks old, 18–22 g) were bought from the Animal Research Center of Wuhan University. The mice were kept in specific pathogen-free (SPF) conditions and provided with sufficient light, air, food, and water (25°C, 60%~70% relative humidity). This research received the approval from the Animal Ethics Committee of Affiliated Nanhua Hospital of the University of South China and abided by The Guide for the Care and Use of Laboratory Animals. Twenty nude mice were classified two groups (ten mice in each group). OS cells transfected with miR-NC and miR-221-3p mimics were injected into the mice. 143B cells (*n* = 10) and Saos2 cells (*n* = 10) were made into single-cell suspension (1 × 10^7^ mL^-1^) and subcutaneously injected into the right back of the mice to establish a xenograft OS model. The tumor volume was determined with vernier caliper on days 7, 14, 21, 28, 35, respectively. After seven days, the mice were executed with euthanasia, and the tumor tissues were stripped off and weighed.

### Immunohistochemistry (IHC)

After conventional paraffin embedding and sectioning (4 μM), the xenograft tumors were subjected to xylene dewaxing, gradient alcohol hydration and blocked with 3% H_2_O_2_ for 10 minutes for endogenous peroxidase inactivation. Then, 0.01mol/L sodium citrate buffer solution was used for microwave repair (pH = 6.0, 15 minutes). After blocking with 5% bovine serum albumin (BSA) for 20 minutes, the primary antibodies (concentration: 1:100) of Ki67 (ab15580), SOCS3 (ab16030), and Vimentin (ab92547) (all from Abcam, MA, USA) were added and incubated overnight at 4°C. On the following day, the goat and anti-rabbit IgG was added dropwise and maintained at RT for 20 minutes, and DAB was employed for coloration after PBS washing. After hematoxylin counterstaining, the tissues were dehydrated, transparentized, and mounted for microscopic examination.

### Statistical analysis

SPSS22.0 software was adopted for data analysis in this experiment. The measurement data were expressed as mean ± standard deviation (x ± s), and the statistical data or percentage (%) were expressed as χ^2^. The comparison between groups was made by one-way ANOVA, while *t* test was used for comparing multiple groups of data. *P* < 0.05 represented statistical significance.

### Ethics statement

Our study was approved by the Ethics Review Board of Affiliated Nanhua Hospital, University of South China.

## RESULTS

### M2-TAMs enhanced the growth and metastasis of OS

**Figure 1:**
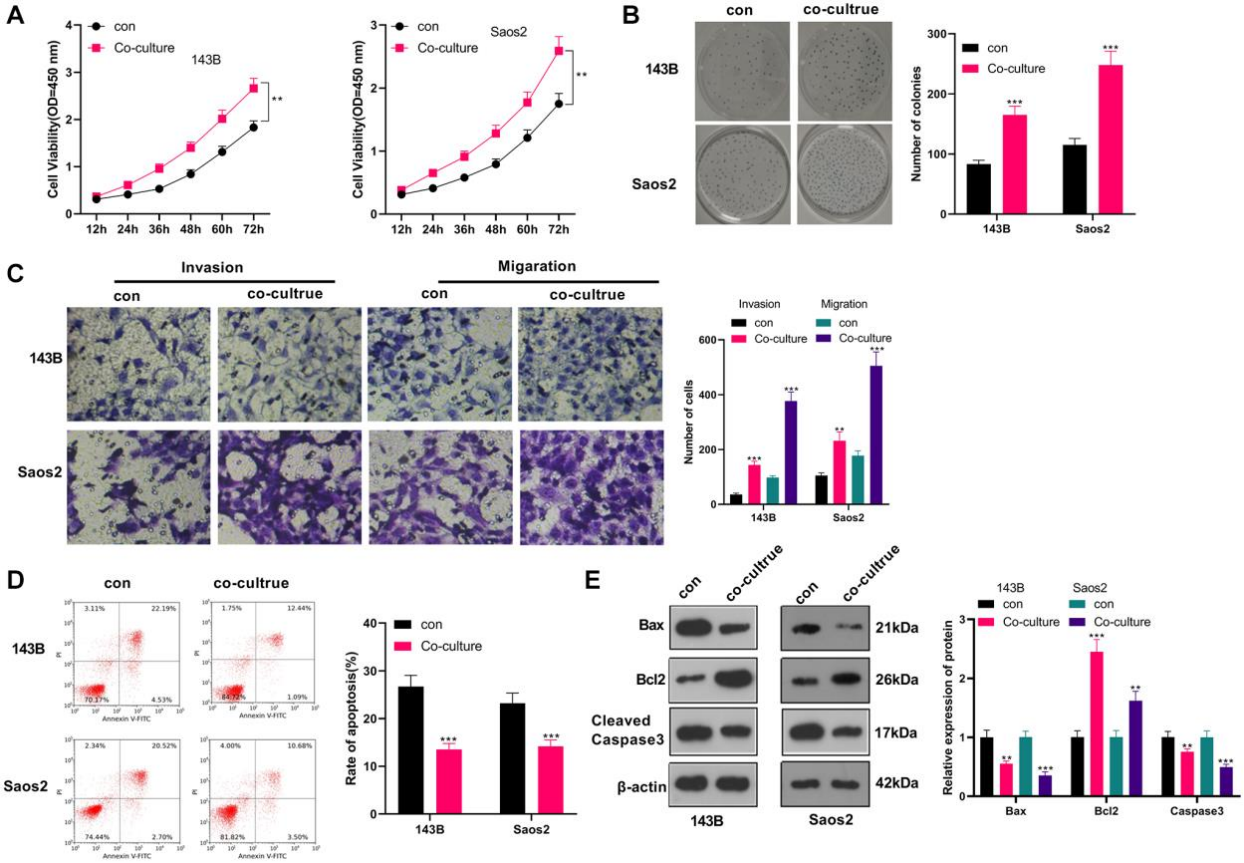
**M2-TAM exosomes strengthened OS growth and metastasis.** Il-4/13-induced M2-TAMs were co-cultured with 143B and Saos2 cells. (**A**) CCK-8 tested cell viability. (**B**) CCK-8 assayed cell proliferation. (**C**) Cell migration and invasion were examined by Transwell assay. (**D**) Cell apoptosis was determined by FCM. (**E**) The profiles of Bax, Bcl2 and Cleaved Caspase-3 were compared by WB. ^*^P < 0.05, ^**^P < 0.01, ^***^P < 0.001 (vs. con group), *N* = 3.

We co-cultured IL-4/13-induced M2-TAMs with human OS cells (143B and Saos2) to probe the influence of M2-TAMs on OS cell growth and metastasis. CCK-8 was adopted to test cell viability at different times (12, 24, 36, 48, 60, and 72 hours). As a result, by contrast with the control group, 143B and Saos2 cell viability in the co-culture group was strengthened (*P* < 0.05, Figure 1A). As per the results displayed in the colony formation assay, 143B and Saos2 cell proliferation was strengthened in the co-culture group (*P* < 0.05, Figure 1B). Moreover, the Transwell assay testified that cell migration and invasion were heightened in the co-culture group (*P* < 0.05, Figure 1C). As indicated by FCM results, the co-culture group had reduced cell apoptosis (vs. the control group) (*P* < 0.05, Figure 1D). Besides, WB results uncovered that Bax and Cleaved Caspase-3 were choked, while Bcl2 was boosted in the co-culture group (vs. the control group) (*P* < 0.05, Figure 1E). These findings illustrated that M2-TAMs amplified 143B and Saos2 cells’ growth, proliferation, migration and invasion and impeded apoptosis.

### OS cells induced M2-TAM activation and up-regulated exosome-derived miR-221-3p

**Figure 2:**
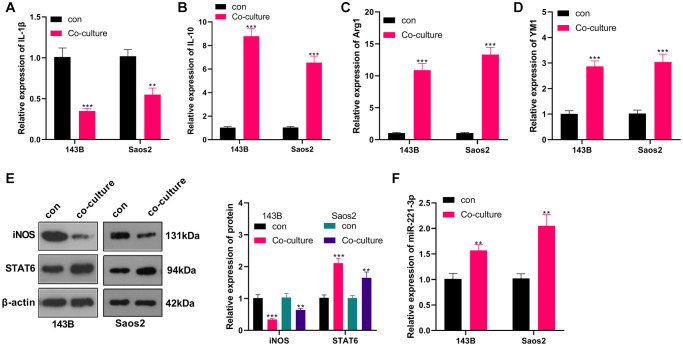
**OS induced M2-TAM activation and boosted exosome-derived miR-221-3p.** M2-TAMs were co-cultured with 143B and Saos2 cells. (**A**–**D**) qRT-PCR assayed the contents of macrophage polarization markers IL-1β, IL-10, Arg1 and YM1; (**E**) The iNOS and STAT6 profiles were verified by WB; (**F**) qRT-PCR was utilized to test the miR-221-3p level. ^**^P < 0.01, ^***^P < 0.001 (VS con group), *N* = 3.

M2-TAMs were co-cultured with OS cells, and we conducted qRT-PCR to verify the profiles of macrophage polarization markers IL-1, IL-10, Arg1, and YM1 in M2-TAMs. The results revealed IL-1β was down-regulated, while IL-10, Arg1 and YM1 levels were augmented in the co-culture group (vs. the control group) (*P* < 0.05, Figure 2A–2D). Meanwhile, WB testified that by contrast with the control group, iNOS expression was abated and STAT6 expression was elevated in the co-cultured group (*P* < 0.05, Figure 2E). Besides, qRT-PCR uncovered that the miR-221-3p profile was heightened in the co-culture group (*P* < 0.05, Figure 2F). Up-regulated miR-221 predicted poorer overall survival of OS patients (Supplementary Figure 1). These findings hinted that the co-culture of 143B and Saos2 cells with TAMs enhanced the “M2” polarization and miR-221-3p profiles.

### Overexpressing miR-221-3p intensified the malignant progression of OS

**Figure 3:**
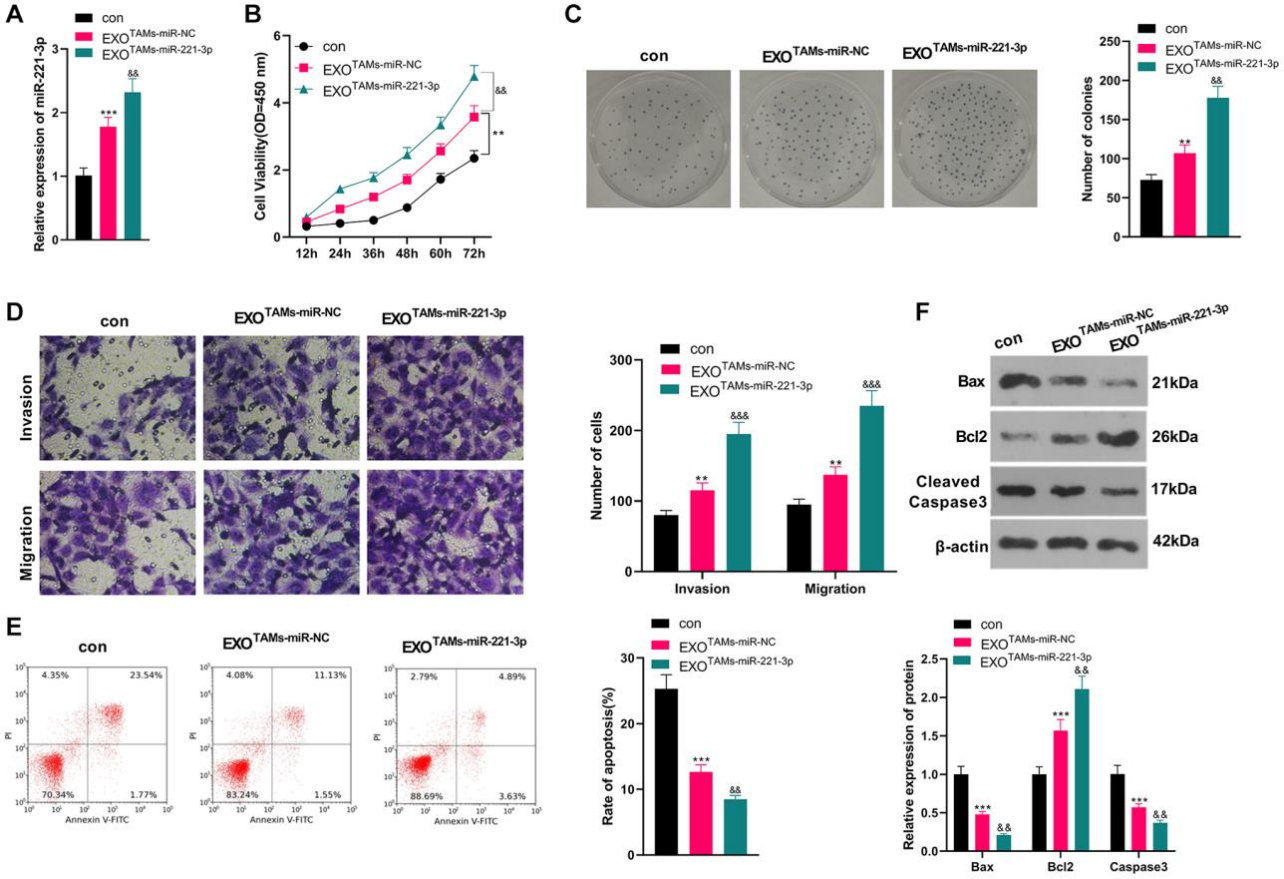
**Overexpressing miR-221-3p aggravated OS.** M2-TAMs were transfected with miR-221-3p mimics. The exosomes in the culture medium of M2-TAMs were isolated and then treated with Saos2 cells. (**A**) qRT-PCR detected the miR-221-3p profile. (**B**) CCK-8 checked cell viability. (**C**) Cell proliferation was tested by the colony formation assay. (**D**) Cell migration and invasion were examined by Transwell assay. (**E**) Cell apoptosis of Saos2 was detected by FCM. (**F**) Bax, Bcl2 and Cleaved Caspase-3 were examined by WB. ^*^P < 0.05, ^**^P < 0.01, ^***^P < 0.001 (vs. con group). ^&^*P* < 0.05, ^&&^*P* < 0.01, ^&&&^*P* < 0.001 (vs. EXO^TAMs+miR-NC^ group), *N* = 3.

We transfected miR-221-3p mimics into M2-TAMs. Then we isolated the exosomes from M2-TAMs and treated Saos2 cells with the exosomes. miR-221-3p was enhanced in THP1 cells as well as the exosomes after miR-221-3p transfection (vs. miR-NC group, Supplementary Figure 2A–2E). qRT-PCR outcomes confirmed that miR-221-3p expression was facilitated in the EXO^TAMs+miR-221-3p^ group (vs. the EXO^TAMs+miR-NC^ group) (*P* < 0.05, Figure 3A). CCK-8 assay confirmed that Saos2 cell viability was enhanced in the EXO^TAMs+miR-221-3p^ group (vs. the EXO^TAMs+miR-NC^ group) (*P* < 0.05, Figure 3B). Additionally, the colony formation assay uncovered that cell proliferation in the EXO^TAMs+miR-221-3p^ group was increased (vs. the EXO^TAMs+miR-NC^ group) (*P* < 0.05, Figure 3C). Besides, the Transwell assay demonstrated that the cell migrative and invasive capacities of the EXO^TAMs+miR-221-3p^ group were strengthened (vs. the EXO^TAMs+miR-NC^ group) (*P* < 0.05, Figure 3D). What’s more, FCM hinted that cell apoptosis was hindered in the EXO^TAMs+miR-221-3p^ group (vs. the EXO^TAMs+miR-NC^ group) (*P* < 0.05, Figure 3E). Also, WB disclosed that the contents of Bax and Cleaved Caspase-3 in the EXO^TAMs+miR-221-3p^ group were impeded, and the Bcl2 profile was boosted (vs. the EXO^TAMs+miR-NC^ group) (*P* < 0.05, Figure 3F). These conclusions suggested that miR-221-3p overexpression facilitated the growth, proliferation, migration and invasion, and hampered apoptosis of Saos2 cells.

### miR-221-3p knockdown in M2-TAM exosomes repressed OS development *in vitro*

**Figure 4:**
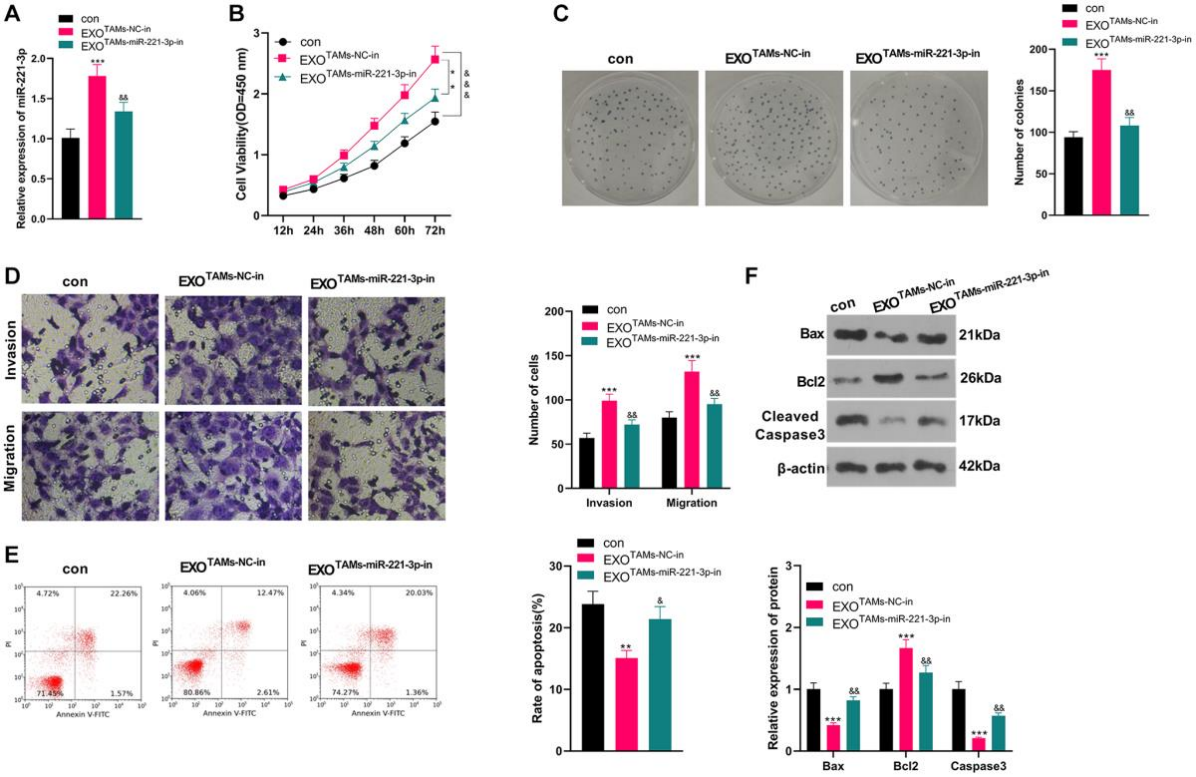
**Knockdown miR-221-3p inhibits the malignant progression of OS.** miR-221-3p-in were transfected into M2-TAMs. The exosomes in the culture medium of M2-TAMs were isolated and then treated with Saos2 cells. (**A**) qRT-PCR verified the miR-221-3p profile. (**B**) CCK-8 monitored cell viability. (**C**) Cell proliferation was monitored by the colony formation experiment. (**D**) Cell migration and invasion were compared by Transwell. (**E**) Apoptosis of Saos2 was detected by FCM. (**F**) Bax, Bcl2 and Cleaved Caspase-3 were examined by WB. ^*^P < 0.05, ^**^P < 0.01, ^***^P < 0.001 (vs. con group). ^&^*P* < 0.05, ^&&^*P* < 0.01, ^&&&^*P* < 0.001 (vs. EXO^TAMs+NC-in^ group), *N* = 3.

We transfected miR-221-3p-in into M2-TAMs. Then we isolated the exosomes from M2-TAMs and treated Saos2 cells with the exosomes. miR-221-3p expression declined in THP1 cells as well as the exosomes after the miR-221-3p-in transfection (vs. NC-in group, Supplementary Figure 2A–2E). qRT-PCR manifested that miR-221-3p expression was hindered in the EXO^TAMs+miR-221-3p-in^ group (vs. the EXO^TAMs+NC-in^ group) (*P* < 0.05, Figure 4A). As indicated by CCK-8 outcomes, by contrast with the TAMs+NC-in group, cell viability of the TAMs+miR-221-3p-in group was hampered (*P* < 0.05, Figure 4B). Moreover, the colony formation assay illustrated that cell proliferation in the EXO^TAMs+miR-221-3p-in^ group was curbed (vs. the EXO^TAMs+NC-in^ group) (*P* < 0.05, Figure 4C). Furthermore, the Transwell assay testified that the EXO^TAMs+miR-221-3p-in^ group had weakened cell migration and invasion (vs. the EXO^TAMs+NC-in^ group) (*P* < 0.05, Figure 4D). Meanwhile, FCM results uncovered that the EXO^TAMs+miR-221-3p-in^ group had intensified cell apoptosis (vs. the EXO^TAMs+NC-in^ group) (*P* < 0.05, Figure 4E). Also, WB confirmed that the concentrations of Bax and Cleaved Caspase-3 in the EXO^TAMs+miR-221-3p-in^ group were boosted, while the Bcl2 level was hindered (vs. EXO^TAMs+NC-in^ group) (*P* < 0.05, Figure 4F). In summary, miR-221-3p knockdown in M2-TAM exosomes impeded Saos2 cell growth, proliferation, migration and invasion, and stimulated apoptosis.

### miR-221-3p targeted SOCS3

**Figure 5:**
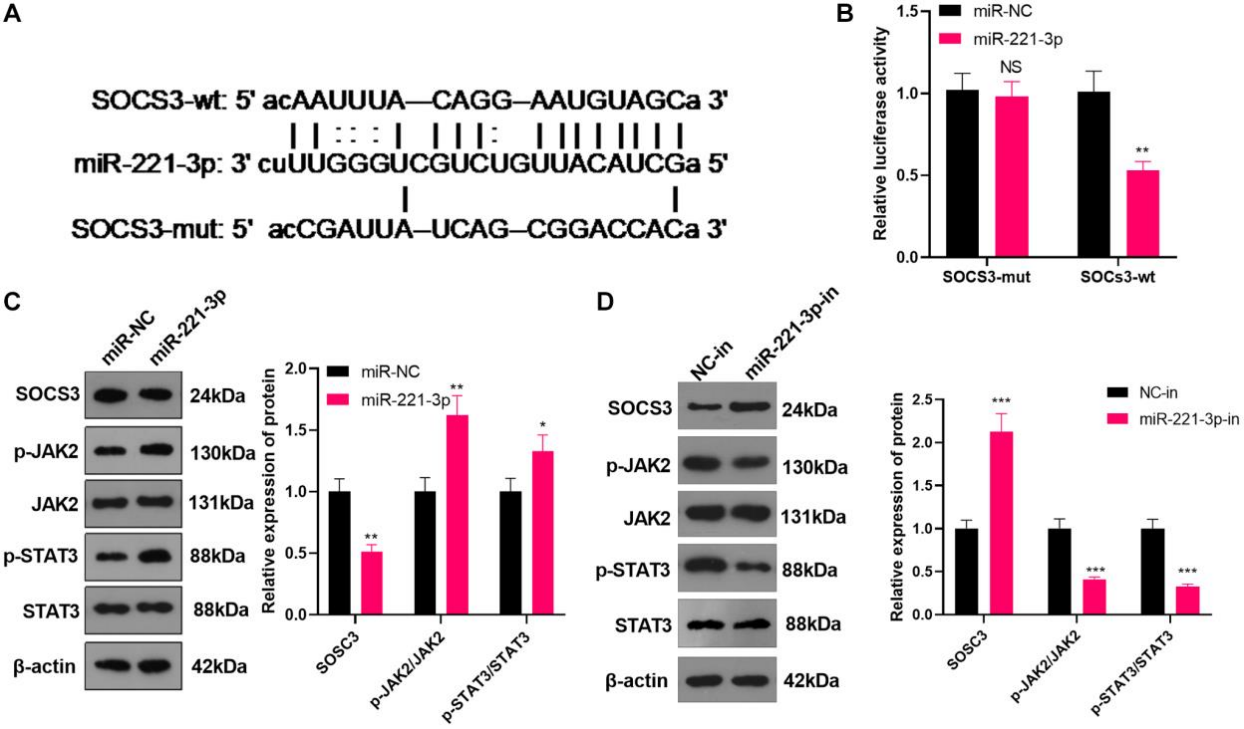
**miR-221-3p targeted SOCS3.** (**A**) The underlying target of miR-221-3p was searched in the Starbase. (**B**) The target link between miR-221-3p and SOCS3 in Saos2 cells was evaluated by the dual-luciferase reporter assay. (**C**–**D**). miR-221-3p mimics, inhibitors or their negative controls were transfected into Saos2 cells. The SOCS3/JAK2/STAT3 expression in Saos2 cells was evaluated by WB. NS *P* > 0.05, ^*^P < 0.05, ^**^P < 0.01 (vs. miR-NC or NC-in group), *N* = 3.

We searched the Starbase (https://web.archive.org/web/20110222111721/http://starbase.sysu.edu.cn/) to verify the association between miR-221-3p and SOCS3 and found that SOCS3 was an important downstream target of miR-221-3p (Figure 5A). Also, the dual-luciferase confirmed that overexpressing miR-221-3p heightened the luciferase activity of SOCS3-3′-UTR-WT THP-1 cells, while it had little impact on mutated SOCS3 (Figure 5B, *P* < 0.05). We transfected Soas2 cells with miR-221-3p mimics or inhibitors and WB was implemented to monitor the SOCS3/JAK2/STAT3 expression and its phosphorylation in Soas2 cells. As a result, SOCS3 expression was impeded, while the phosphorylation of JAK2/STAT3 was elevated in the miR-221-3p group (vs. the miR-NC group) (Figure 5C, *P* < 0.05). On the opposite, transfection of miR-221-3p-in boosted SOCS3 and repressed the phosphorylation of JAK2/STAT3 in Soas2 cells (vs. the NC-in group) (Figure 5D, *P* < 0.05). Hence, miR-221-3p targeted SOCS3 and activated JAK2/STAT3 in OS cells.

### Overexpressing miR-221-3p curbed SOCS3 and activated JAK2/STAT3

**Figure 6:**
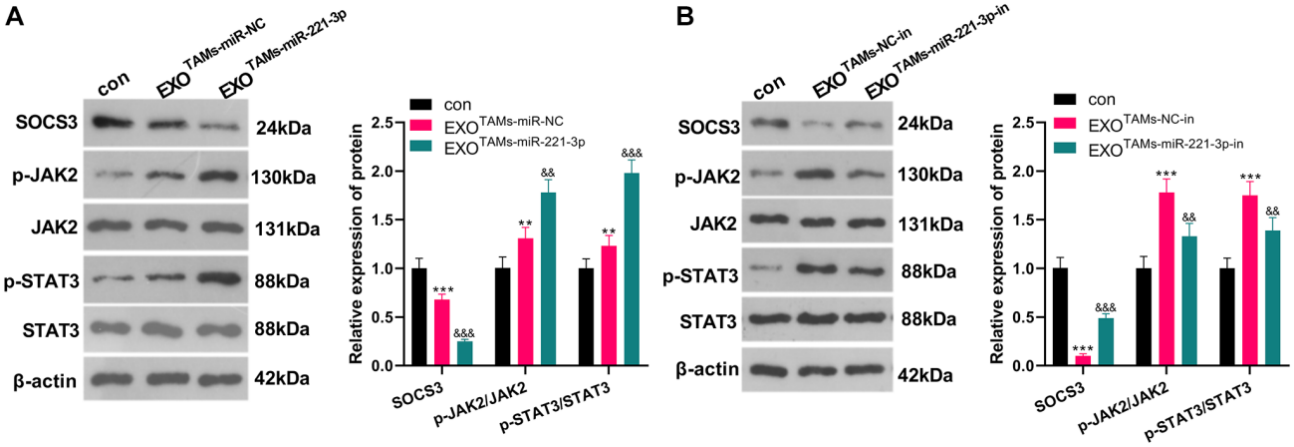
**miR-221-3p overexpression curbed SOCS3 and boosted JAK2/STAT3.** miR-221-3p mimics or inhibitors were transferred into M2-TAMs. The exosomes in the culture medium of M2-TAMs were isolated and then treated with Saos2 cells. (**A**–**B**) WB was used for analyzing the SOCS3/JAK2/STAT3 pathway expression. ^**^P < 0.01, ^***^P < 0.001 (vs. con group). ^&&^*P* < 0.01, ^&&&^*P* < 0.001 (vs. EXO^TAMs+miR-NC^ group or EXO^TAMs+NC-in^ group), *N* = 3.

miR-221-3p mimics, inhibitors and their negative controls were respectively transfected into M2-TAMs. Then we isolated the exosomes from M2-TAMs and treated Saos2 cells with the exosomes. The SOCS3/JAK2/STAT3 axis was detected by WB. As a result, SOCS3 was down-regulated, and the JAK2/STAT3 phosphorylation was strengthened in the EXO^TAMs+miR-221-3p^ group (vs. the EXO^TAMs+miR-NC^ group) (Figure 6A, *P* < 0.05). On the contrary, SOCS3 level was enhanced and the JAK2/STAT3 phosphorylation was repressed after EXO^TAMs+miR-221-3p-in^ treatment (vs. the EXO^TAMs+NC-in^ group) (Figure 6B, *P* < 0.05). These findings indicated that miR-221-3p enriched by M2-TAM exosomes dampened the SOCS3 expression and activated JAK2/STAT3 in OS cells.

### Inhibition of JAK2/STAT3 reversed the oncogenic function of miR-221-3p

**Figure 7:**
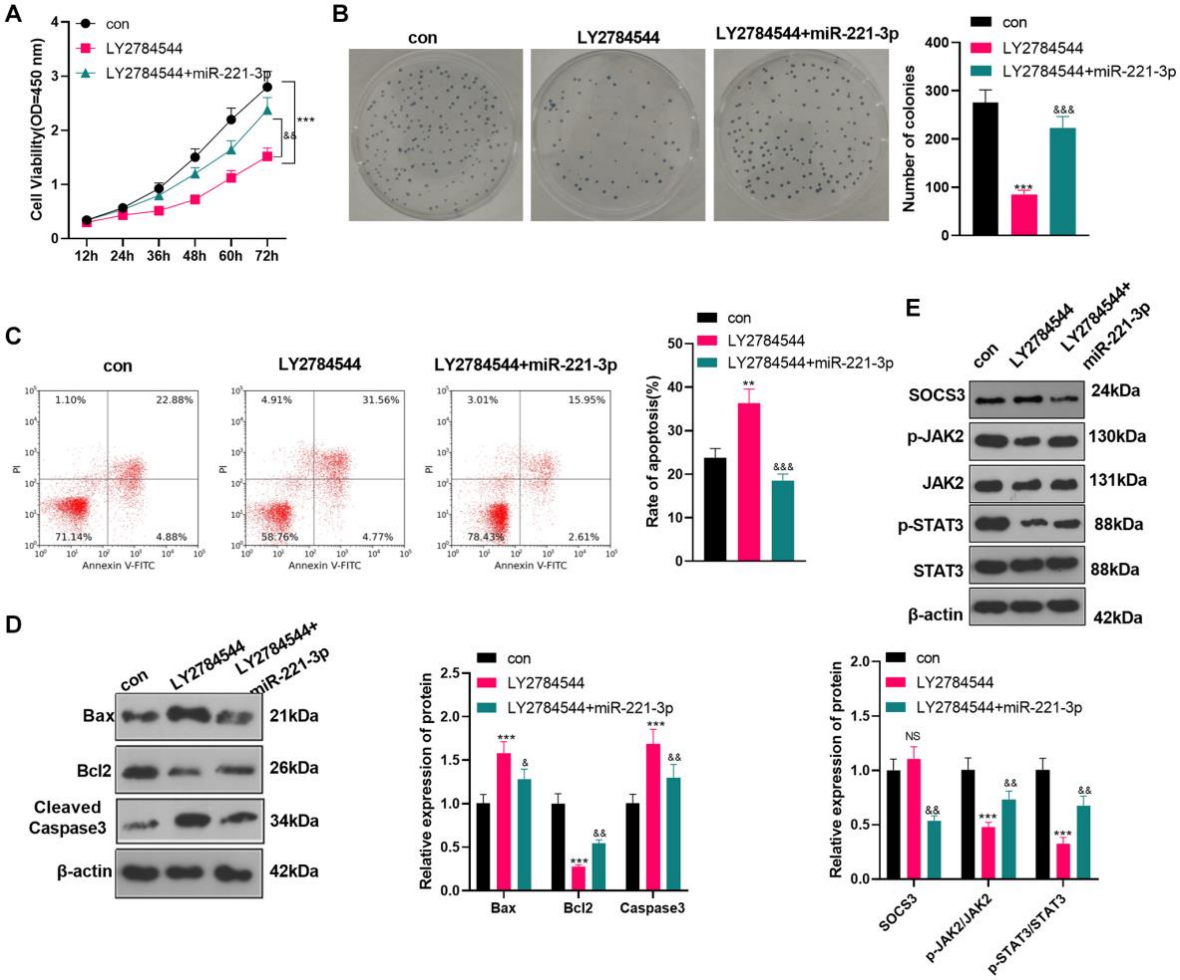
**Inhibiting JAK2/STAT3 reversed the oncogenic property of miR-221-3p.** LY2784544 (3 nM) was applied to treat Saos2 cells, which were transfected with miR-221-3p mimics. (**A**) CCK-8 tested cell viability. (**B**) Cell proliferation was monitored by the colony formation assay. (**C**) FCM was utilized to detect cell apoptosis. (**D**) The profiles of Bax, Bcl2 and Cleaved Caspase-3 were assessed by WB. (**E**) WB was conducted to detect the SOCS3/JAK2/STAT3 profile and its phosphorylation. NS*P*>0.05, ^**^P < 0.01, ^***^P < 0.001 (vs. con group), ^&^*P* < 0.05, ^&&^*P* < 0.01, ^&&&^*P* < 0.001 (vs. LY2784544 group), *N* = 3.

To confirm the role of JAK2/STAT3 in OS cells, we treated OS cells (Saos2) with the JAK2/STAT3 pathway inhibitor LY2784544. Then miR-221-3p mimics were transferred into OS cells. CCK-8 and colony formation assay showed that Saos2 cells’ viability and proliferation in the LY2784544 group were decreased (vs. the control group). However, by contrast with the LY2784544 group, the LY2784544 +miR-221-3p group showed elevated cell viability (Figure 7A–7B, *P* < 0.05). Also, FCM testified that cell apoptosis was heightened in the LY2784544 group (vs. the control group) and was abated in the LY2784544 +miR-221-3p group (vs. the LY2784544 group) (Figure 7C, *P* < 0.05). Moreover, WB verified that Bax and Cleaved Caspase-3 expression was amplified, while Bcl2 was choked in the LY2784544 group (vs. the control group). On the contrary, opposite results were monitored in the LY2784544 +miR-221-3p group (vs. the LY2784544 group) (Figure 7D, *P* < 0.05). Besides, the SOCS3/JAK2/STAT3 pathway expression was detected by WB. It turned out that the activation of JAK2/STAT3 in the LY2784544 group was decreased, but the SOCS3 level remained unchanged (vs. the control group). In contrast, the JAK2/STAT3 phosphorylation in the LY2784544 +miR-221-3p group was enhanced, but the SOCS3 level was lowered (vs. the LY2784544 group) (Figure 7E, *P* < 0.05). These findings demonstrated that inhibiting JAK2/STAT3 reduced the malignant behaviors of OS cells, and miR-221-3p reversed the anti-tumor function of LY2784544.

### miR-221-3p boosted tumor growth in tumor-bearing mice

**Figure 8:**
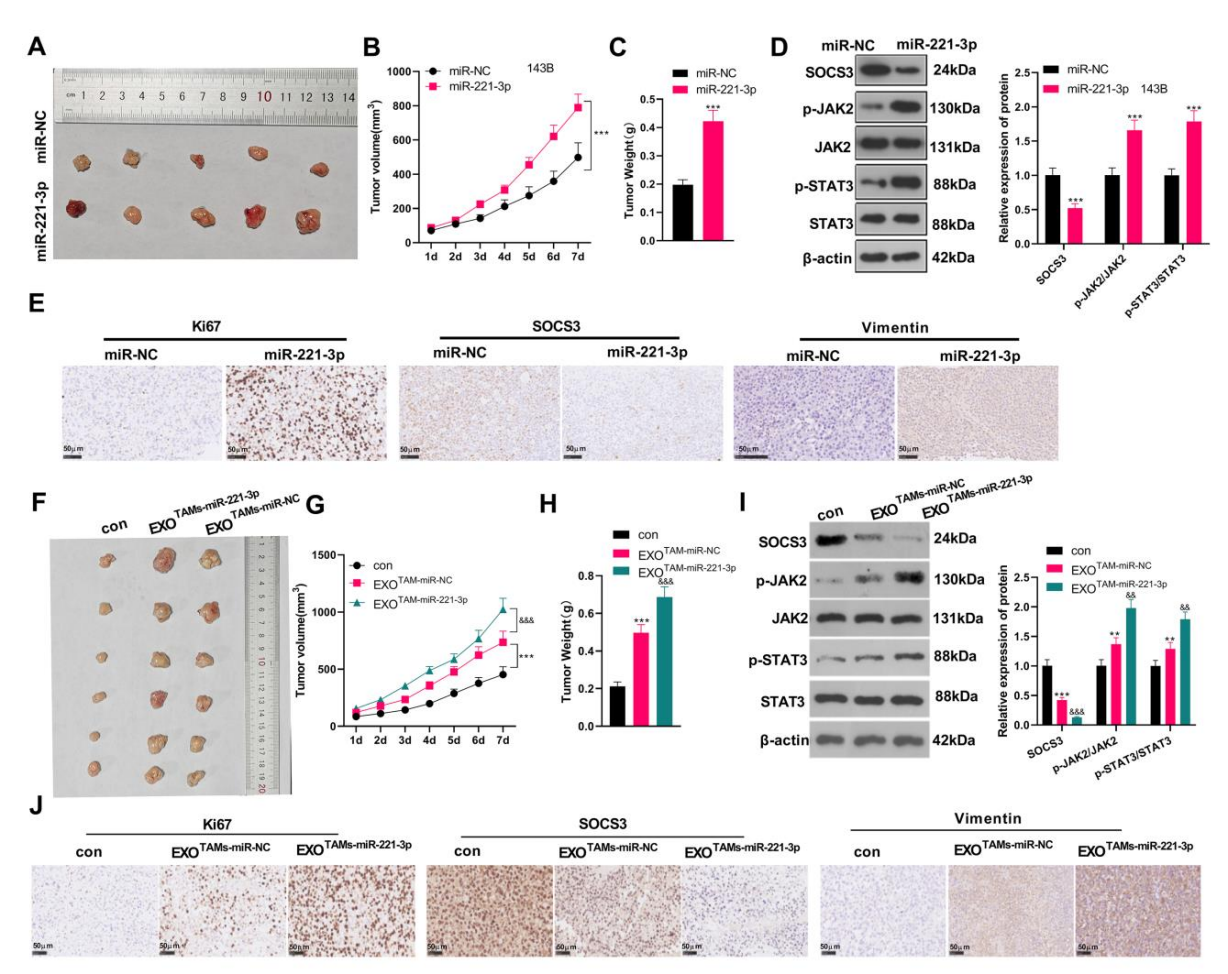
**miR-221-3p boosted tumor growth in tumor-bearing mice.** miR-221-3p mimics were subcutaneously injected into tumor-bearing mice to observe tumor growth. (**A**) The tumor. (**B**) The tumor volume. (**C**) The tumor weight. (**D**) WB examined the SOCS3/JAK2/STAT3 pathway expression and its phosphorylated level in mouse tumor tissues. (**E**) IHC detected the positive numbers of Ki67, SOCS3 and Vimentin. ^***^P < 0.001 (vs. miR-NC group), *N* = 5. miR-221-3p mimics were transfected into M2-TAMs. The exosomes in the culture medium of M2-TAMs were isolated and then subcutaneously injected into tumor-bearing mice to observe tumor growth. (**F**) The tumor tissue. (**G**) The tumor volume. (**H**) The tumor weight. (**I**) WB examined SOCS3/JAK2/STAT3 expression and its phosphorylated level in mouse tumor tissues. (**J**) IHC detected the positive numbers of Ki67, SOCS3 and Vimentin. ^**^P < 0.01, ^***^P < 0.001 (vs. con group); ^&&^*P* < 0.01, ^&&&^*P* < 0.001 (vs. EXO^TAMs+miR-NC^ group), *N* = 5.

We subcutaneously injected miR-221-3p mimics or M2-TAM exosomes overexpressing miR-221-3p into tumor-bearing mice and observed the tumor growth *in vivo.* We found that by contrast with the miR-NC group, the tumor volume and mass of the miR-221-3p group increased significantly (Figure 8A–8C, *P* < 0.05). WB and IHC were employed to verify the SOCS3/JAK2/STAT3 expression in the tumor tissues of mice. The results revealed that SOCS3 was down-regulated, while Ki-67 positive cells were increased and JAK2/STAT3 was facilitated in the miR-221-3p group (vs. the miR-NC group) (Figure 8D–8E, *P* < 0.05). Additionally, the EXO^TAMs-miR-221-3p^ injection promoted tumor growth and increased tumor weight (vs. EXO^TAMs-miR-NC^ group, Figure 8F–8H). The results of ICH and WB suggested that EXO^TAMs-miR-221-3p^ injection reduced SOCS3 and promoted JAK2/STAT3 pathway activation (vs. EXO^TAMs-miR-NC^ group, Figure 8I–8J). interestingly, EXO^TAMs-miR-221-3p^ injection also enhanced Ki67 positive cell rate (vs. EXO^TAMs-miR-NC^ group, Figure 8J). In summary, miR-221-3p exerted oncogenic effects *in vivo*.

## DISCUSSION

OS is a frequent primary bone malignancy, mostly occurring in children and adolescents under 25 years old [19–20]. Because OS is highly metastatic and aggressive, the patient's prognosis is poor, and it is easy to relapse, which seriously affects the survival time of the patients [21]. With the continuous exploration of OS, we have found that various miRNAs are involved in regulating OS [22–24]. This article intends to probe the impact of miR-221-3p on OS cells’ growth and invasion in M2-polarized TAM exosomes.

In recent years, mounting studies have revealed that exosome-derived miRNAs regulate various cancers, such as hepatocellular carcinoma [25]. TAM-secreted exosome-derived miRNAs directly or indirectly induce tumor metastasis by regulating molecular levels in the tumor microenvironment and transforming distant mesenchymal cells, influencing OS progression [12]. Multiple studies have revealed that miR-143 represses OS. miR-143 promotes U2-OS and MG-63 cells’ apoptosis and dampens migration and invasion via Cleaved Caspase-3 activation by targeting Bcl-2 [26]. TGF-β1 inhibits the expression of miR-143 through the Smad 2/3-dependent pathway, and the Boyden chamber experiment verified that OS cell migration and invasion are promoted [27]. Besides, Gong L et al. held that exosome-derived miR-675 is overexpressed in metastatic OS cells, and miR-675 targets CALN1 to enhance the migration and invasion of metastatic OS cells [28]. Taken together, miRNAs play different roles in OS due to their different targeted downstream pathways and molecules. In this experiment, exosome-derived miR-221-3p boosts OS cells’ growth and metastasis.

SOCS3 belongs to the SOCS family, and a variety of cytokine receptors and hormones affect the SOCS3 expression by regulating the phosphorylation of JAK2 and STAT in different types of cells and tissues [29–30]. SOCS3 reversely regulates the JAK2/STAT3 axis. It is abnormally activated by the JAK/STAT pathway, which dampens the JAK expression and thus represses STAT3 phosphorylation. Previous reports have stated that the SOCS3/JAK2/STAT3 axis modulates the occurrence and development of various cancers [31–33]. Du YX et al. reported that CIRC-ANKIB1 up-regulates miR-19b, inhibits the expression of the downstream gene SOCS3, and phosphorylates STAT3, thus heightening the growth and invasion of OS cells [34]. Besides, Simvastatin dose-dependently enhances the SOCS3 profile in OS UMR-106 cells and reduces the phosphorylation of the JAK2/STAT5 pathway, thereby inhibiting OS cells’ growth, proliferation, migration and invasion [35]. The function of SOCS3 in negatively JAK/STAT and affecting the growth and proliferation of OS cells has been testified in the past.

Here, we focused on the mechanism of miR-221-3p derived from M2-TAMs in the progression of OS. We found miR-221-3p targeted SOCS3, which was consistent with precious researches [36–38] and confirmed in several studies [1–3]. Our *in-vitro* and *in-vivo* data confirmed that SOCS3 was repressed following miR-221-3p overexpression. Therefore, we revealed that “M2”-TAM enhances the malignant progression of OS via inducing miR-221-3p up-regulation in OS cells, which then targets SOCS3 and activates JAK2/STAT3 in OS cells, thereby inducing enhanced proliferation and metastasis of OS cells. However, this miR-221-3p-SOCS3 axis is not a novel regulatory axis in cancer, but it was not confirmed in OS by a previous study. Therefore, we believed this study gives a new reference in OS evolvement.

**Figure 9:**
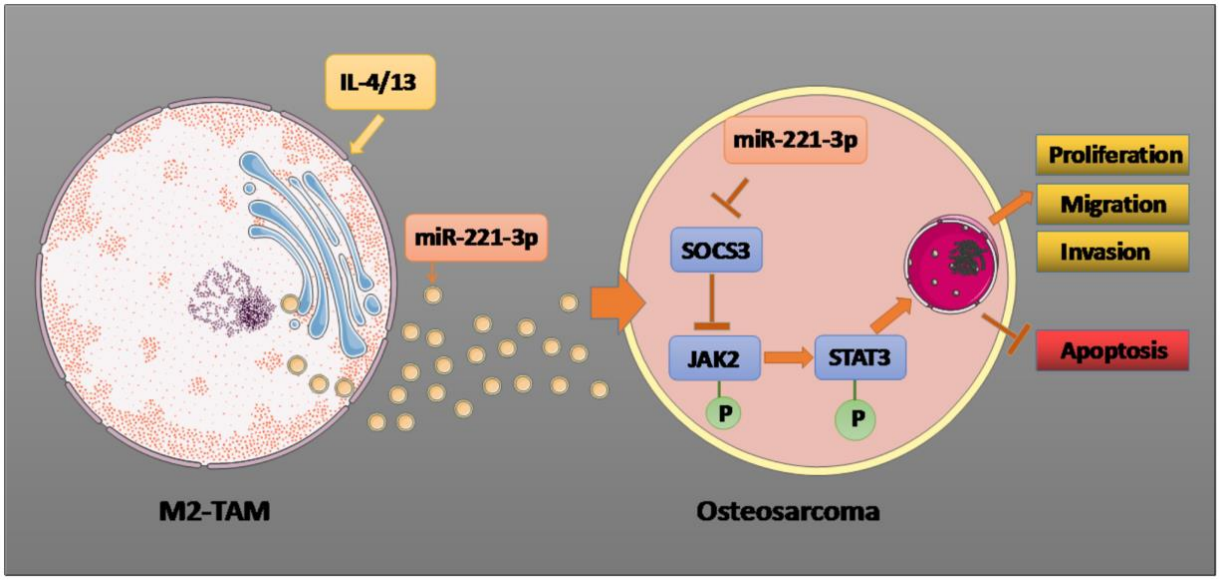
**Graphical abstract.** Il-4/13 induces M2-TAMs, and miR-221-3p produced by M2-TAM exosomes targets and dampens SOCS3, thus phosphorylating JAK2/STAT3, stimulating proliferation, migration and invasion of OS cells, and hampering cell apoptosis. The arrow indicates the activation effect, and the T symbol represents the inhibitory effect.

In a nutshell, this research confirmed that miR-221-3p produced by IL-4/13-induced M2-TAM exosomes significantly enhances the malignant progression of OS cells. Further mechanism researches uncovered that exosomal miR-221-3p targets the downstream SOCS3 molecules and up-regulates JAK2/STAT3 to affect OS progression (Figure 9). Overall, this article brings a new idea for the clinical therapy of OS, and miR-221-3p is an underlying target for treating OS. However, miR-221-3p’s function in regulating the JAK2/STAT3 pathway in OS patients has not been verified clinically. Further verification will be conducted in subsequent clinical studies.

## Supplementary Materials

Supplementary Figures
