# Supplementary material for: miRNA-221-3p derived from M2-polarized tumor-associated macrophage exosomes aggravates the growth and metastasis of osteosarcoma through SOCS3/JAK2/STAT3 axis
Source: Aging (Albany NY). 2021 Aug 13;13(15):19760–75. doi: 10.18632/aging.203388 (PMC8386545; doi:10.18632/aging.203388)
Supplement: Supplementary Figures [file aging-13-203388-s001.pdf]

SUPPLEMENTARY FIGURES

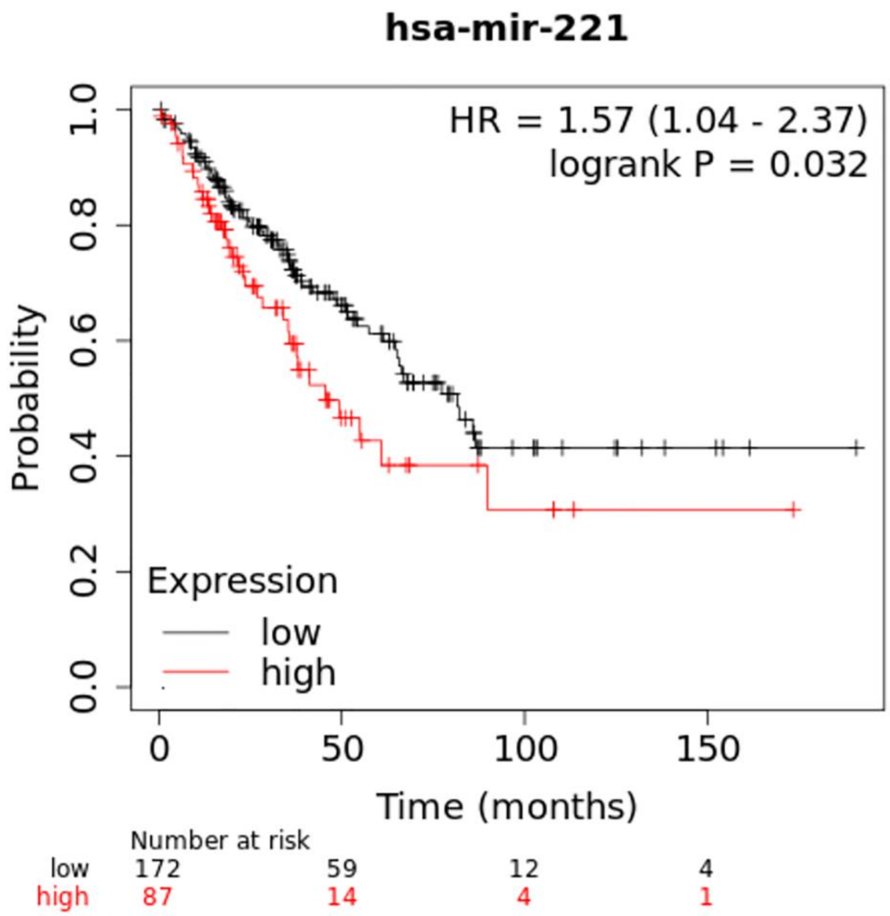

Supplementary Figure 1. The online database Kaplan-Meier Plotter (http://kmplot.com/analysis/) was used for analyzing the link between the miR-221 level and the overall survival of sarcoma patients.

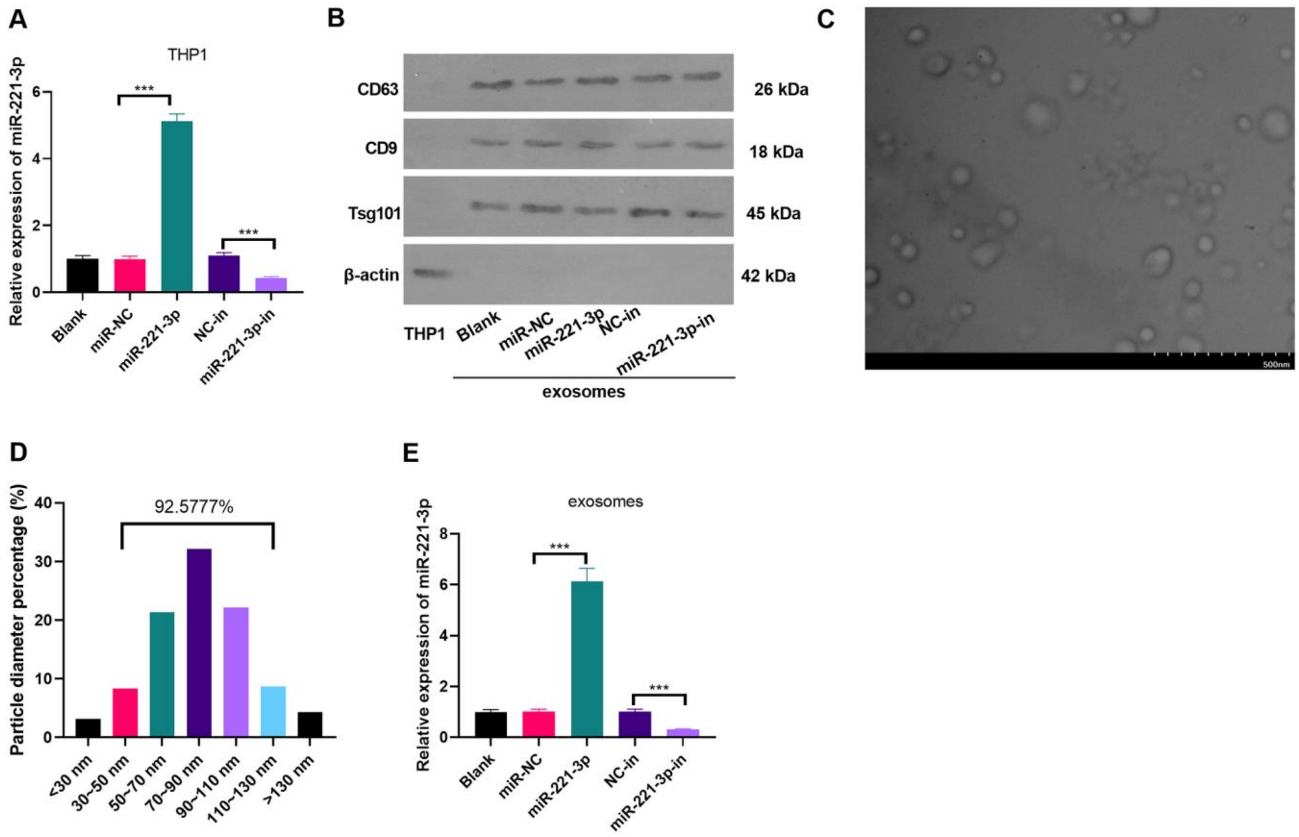

Supplementary Figure 2. (**A**) THP1 cells were transfected with miR-221-3p mimics or miR-221-3p-in, then RT-PCR was adopted for checking miR-221-3p levels in the cells. (**B**) WB was used for detecting CD63, CD9, Tsg101 and β-actin in THP1 cells or the exosomes isolated in the culture medium. (**C**) SEM was used for observing the exosomes. (**D**) The diameter range of the isolated exosomes. (**E**) RT-PCR was used for detecting miR-221-3p levels in the exosomes.
